# Human Neutrophils Kill *Bacillus anthracis*

**DOI:** 10.1371/journal.ppat.0010023

**Published:** 2005-11-11

**Authors:** Anne Mayer-Scholl, Robert Hurwitz, Volker Brinkmann, Monika Schmid, Peter Jungblut, Yvette Weinrauch, Arturo Zychlinsky

**Affiliations:** 1 Department of Cellular Microbiology, Max-Planck-Institute for Infection Biology, Berlin, Germany; 2 Biochemistry Core Facility, Max-Planck-Institute for Infection Biology, Berlin, Germany; 3 Microscopy Core Facility, Max-Planck-Institute for Infection Biology, Berlin, Germany; 4 Protein Analysis Core Facilities, Max-Planck-Institute for Infection Biology, Berlin. Germany; 5 Department of Microbiology, New York University School of Medicine, New York, New York, United States of America; The Salk Institute for Biological Studies, United States of America

## Abstract

*Bacillus anthracis* spores cause natural infections and are used as biological weapons. Inhalation infection with *B. anthracis,* the etiological agent of anthrax, is almost always lethal, yet cutaneous infections usually remain localized and resolve spontaneously. Neutrophils are typically recruited to cutaneous but seldom to other forms of anthrax infections, raising the possibility that neutrophils kill *B. anthracis.* In this study we infected human neutrophils with either spores or vegetative bacteria of a wild-type strain, or strains, expressing only one of the two major virulence factors. The human neutrophils engulfed *B. anthracis* spores, which germinated intracellularly and were then efficiently killed. Interestingly, neutrophil killing was independent of reactive oxygen species production. We fractionated a human neutrophil granule extract by high-performance liquid chromatography and identified α-defensins as the component responsible for *B. anthracis* killing. These data suggest that the timely recruitment of neutrophils can control cutaneous infections and possibly other forms of *B. anthracis* infections, and that α-defensins play an important role in the potent anti-*B. anthracis* activity of neutrophils.

## Synopsis

*Bacillus anthracis* is the bacterium that causes anthrax, a disease that can occur through natural infections and also through intentional release. *B. anthracis* makes spores, which are in a dormant state, similar to seeds of a plant, and are extremely resistant to the environment. *B. anthracis* spores can infect through the skin or the lung. Lung infections disseminate through the body and are lethal. In contrast, skin infections often remain localized, and patients survive even without treatment. It is not well understood why these bacteria cause a localized infection through the skin and a lethal disease through the lung.

Little is known about how *B. anthracis* is controlled. Neutrophils are the first white blood cells recruited to a site of infection and are specialized in killing microbes. Previous studies show that neutrophils are abundant in the skin form, but not in the lung form of anthrax. The researchers report that human neutrophils can take up *B. anthracis* spores. Once inside, the spores germinate to form vegetative bacteria. The vegetative bacteria are extremely susceptible to neutrophil-killing mechanisms. The *B. anthracis* virulence factors (molecules that make bacteria cause diseases) manipulate other human cells but do not deter neutrophils. *B. anthracis* is indeed exquisitely sensitive to the neutrophil protein α-defensin. These data support a new model where *B. anthracis* skin, but not lung, infections are controlled by the antimicrobial activity of neutrophils.

## Introduction

The Gram-positive bacterium *Bacillus anthracis* infects through intradermal inoculation, ingestion, or inhalation of spores. Spores are dormant forms of *B. anthracis* and are extremely resistant to environmental stress. In the current model of respiratory infections, the spores are first taken up by macrophages where they germinate and become vegetative bacteria [1]. Macrophages then transport the bacteria to the regional lymph nodes [2]. In these organs, the bacteria escape from the macrophage and spread through the lymphatics and blood stream causing massive septicemia. Vegetative *B. anthracis* express two essential virulence factors: the tripartite anthrax toxin and the poly-γ-D glutamic acid capsule [3]. The toxin-protective antigen binds to its specific receptor and translocates the edema and lethal factors into the cytosol [4]. Edema factor is an adenylate cyclase that causes tissue edema [5], whereas lethal factor is a metalloprotease that inactivates mitogen-activated protein kinase-kinase and provokes cell death [6].

Neutrophils are a vital component of the acute inflammatory response and play a key role in the resolution of microbial infections. They are terminally differentiated cells, incapable of cell division, and synthesize very low levels of RNA and protein. Neutrophils engulf microbes into a phagosome that fuses with intracellular granules to form a phagolysosome. In the phagolysosome the bacteria are killed through the interaction of reactive oxygen species (ROS) [7] and oxygen-independent mediators such as enzymes and antimicrobial peptides [8]. Antimicrobial peptides are predominantly cationic and are thought to permeabilize the bacterial membrane and lyse microbes [9].

Inhalation *B. anthracis* infections result in sepsis and death, while cutaneous anthrax almost always remains localized. Interestingly, in untreated cutaneous cases, neutrophils surround the necrotic, bacteria-containing tissue [10], whereas neutrophil infiltration is rarely seen in the lung during inhalation anthrax [11]. Although there are reports of pulmonary infiltration in *B. anthracis* infections [12], they were thought to be due to preexisting lesions [13,14]. Pigs and dogs also develop a cutaneous form when infected subcutaneously. This form is reminiscent of human cutaneous anthrax and is accompanied by massive neutrophil infiltrations [13]. Also, neutrophil recruitment is found in the lungs of pulmonary infected dogs and pigs that survive high infection doses [14]. Therefore, we hypothesized that neutrophils can kill *B. anthracis.*

## Results/Discussion

### Human Neutrophils Kill *B.anthracis* Spores Independent of Reactive Oxygen Species

**Figure 1:**
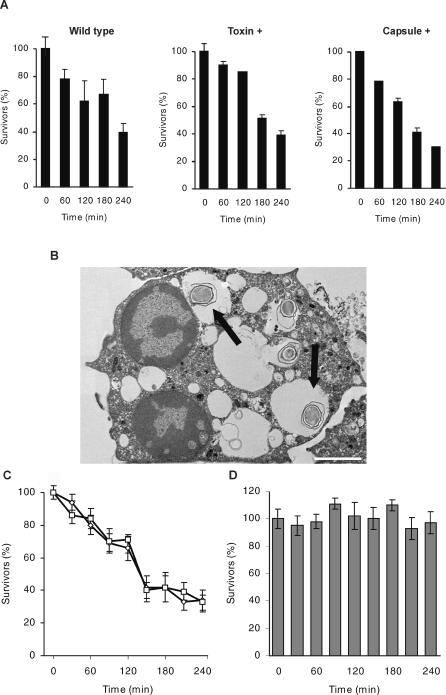
Human Neutrophils Kill *B. anthracis* Spores Independently of Reactive Oxygen Species **(**A) Human neutrophils kill *B. anthracis* spores. Activated neutrophils were infected with spores generated either from the wild-type or toxin- or capsule-producing strains at an MOI (spore: neutrophil) of 5:1 (wild-type) or 1:1 (toxin- and capsule-producers). Colony forming units (CFUs) were counted at indicated time points. (B) TEM of activated neutrophils infected with wild-type spores (arrow) after 30 min incubation. Bar indicates 2 μm. (C) ROS are not required for killing of *B. anthracis* spores. Activated neutrophils were infected with spores (capsule producer) in presence (□) or absence (⋄) of NADPH oxidase inhibitor. (D) hNGE does not kill *B. anthracis* spores. Spores from the toxin-producing strain were incubated in 50% hNGE or in buffer for the indicated time points. The CFU was determined by serial dilution. Survival of 100% refers to the number of bacteria present in the buffer control. Error bars indicate the SD from three experiments.

We tested wild-type *B. anthracis* and strains expressing only the toxin or the capsule. We incubated neutrophils with a wild-type strain at a multiplicity of infection (MOI) of 5:1 (spores: neutrophils) and measured bacterial viability at different times. Activated neutrophils killed 60% of spores after 3 h, and at 4 h only 30% of the spores survived. Similar results were obtained from infections with the toxin- or capsule-producing strains (Figure 1A). Spores of wild-type (Figure 1B), as well as the other two strains (unpublished data), were phagocytosed by activated neutrophils. In the absence of serum, around 50% of the spores were found intracellularly 30 min post-infection as determined by counting spores in electron microscopy samples. These data are in agreement with Welkos et al. [15] who showed that murine neutrophils can kill toxin-positive and capsule-negative spores, albeit with lower efficiency, than we observed with human neutrophils. To ensure that spores and not vegetative bacteria were ingested, the killing efficiency was tested in serum-free media since some sera induce germination [16].

Neutrophils kill microbes through oxygen-dependent and independent mechanisms. ROS are generated by the nicotinamide adenosine dinucleotide phosphate (NADPH) oxidase and are important in some bacterial infections. To determine the role of ROS in the killing of *B. anthracis,* we inactivated neutrophil NADPH oxidase with diphenyleneiodonium (DPI) [17] (Figure S1), and found that the antimicrobial activity was not dependent on ROS (Figure 1C). We then tested whether antimicrobial proteins were effective against *B. anthracis* by incubating spores with a human neutrophil extract enriched in granule proteins (hNGE). Even at very high hNGE concentrations the spores remained viable throughout a 4-h incubation (Figure 1D) and did not germinate during this time (unpublished data). These findings prompted us to test whether spores germinate in neutrophils and whether the vegetative forms are then susceptible to neutrophil killing.

### *B. anthracis* Spores Germinate within Human Neutrophils

**Figure 2:**
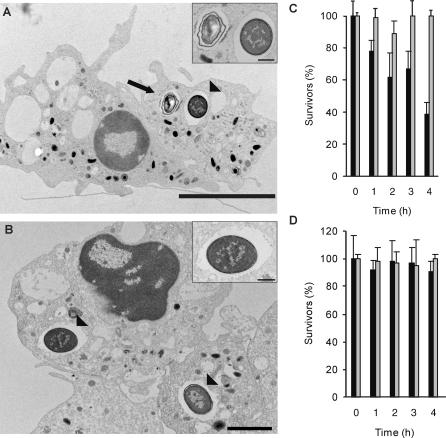
*B. anthracis* Spores Germinate within Human Neutrophils (A) TEM of neutrophil infected with spores of the toxin-producing strain for 90 min at an MOI of 1:1. At this time point both spores (arrow) and germinated bacteria (arrowhead) were present (see inset for amplification). (B) Neutrophil infected for 180 min. At this time point neutrophils contained mostly germinated bacteria (arrowheads). Bars in (A) indicate 5 μm, in (B) 2 μm, and in insets 500 nm. (C) Phagocytosis of *B. anthracis* spores is essential for neutrophil killing. Phagocytosis of activated neutrophils was inhibited with cytochalasin D (grey bars). Cells were infected with wild-type strain at an MOI of 5:1. Controls were PMA-activated cells not treated with cytochalasin D (black bars). (D) *B. anthracis* spores do not germinate in the presence of neutrophils. PMA-activated neutrophils were pretreated with cytochalasin D in the presence (black bars) or absence (grey bars) of penicillin G (25μg/ml). The cells were infected with wild-type strain spores at an MOI of 5:1 and incubated for the given time points. The CFU was determined by serial dilution. Survival of 100% refers to the number of spores present in the controls lacking penicillin G. Error bars indicate the SD from three experiments.

Neutrophils were infected with spores, and the germination of *B. anthracis* in the cells was analyzed by transmission electron microscopy (TEM). At 90 min post-infection, newly germinated vegetative bacteria were observed for the first time (Figure 2A). In contrast, spore uptake was recorded after 30 min. By 180 min, neutrophils contained mostly germinated bacteria (Figure 2B). The spores and vegetative bacteria were found in membrane-bound vacuoles, most likely phagosomes. Intriguingly, the germination process in both neutrophils and macrophages [1] does not seem synchronized, as spores were found throughout the infection period. Whether the spores remain unresponsive to cell-specific germinants, remains to be examined.

To confirm that germination occurred intracellularly, we prevented neutrophil phagocytosis by incubating activated neutrophils with cytochalasin D, an inhibitor of actin polymerization. Inhibition of phagocytosis completely abrogated the sporicidal activity of the neutrophils (Figure 2C), supporting the electron microscopy data that spores germinate within the cells prior to killing. To further verify that spores did not germinate extracellularly, we incubated spores in the presence of cytochalasin D-treated neutrophils and added an antibiotic to kill any newly developed vegetative cells. There was no difference in the counts of spores incubated with cytochalsin D-treated neutrophils in the absence or presence of antibiotics (Figure 2D). These data indicate that degranulating neutrophils do not induce extracellular germination.

### Human Neutrophils Efficiently Kill Vegetative *B. anthracis*

**Figure 3:**
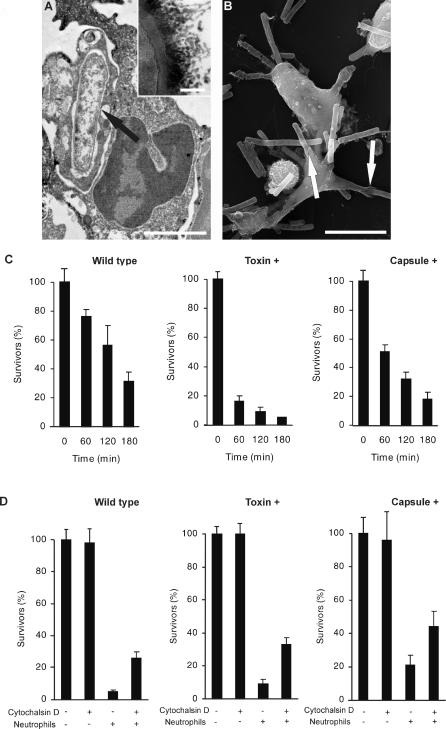
Human Neutrophils Efficiently Kill Vegetative *B. anthracis* Intra- and Extracellularly (A) TEM of neutrophils infected with wild-type *B. anthracis* (arrow) at an MOI of 1:1. Bar = 1μm. Inset indicates the inoculum. Bar = 100 nm. (B**)** Scanning electron microscopy of neutrophils infected with vegetative form of the toxin-producing strain for 30 min. Phagocytosed bacteria shown by arrow. Neutrophils efficiently engulf *B. anthracis* despite their size. Bar = 5μm. **(**C) Neutrophils kill *B. anthracis* efficiently. Activated neutrophils were infected with wild-type, capsule-, or toxin-expressing vegetative bacteria for indicated time points at an MOI of 10:1. (D) *B. anthracis* capsule and toxin are not antiphagocytic. Activated neutrophils were pretreated with the phagocytosis inhibiting cytochalsin D (10 μg/ml), infected with wild-type, capsule-, or toxin-expressing vegetative bacteria at an MOI of 1:1, and incubated for 30 min.

To determine whether vegetative *B. anthracis* are killed by neutrophils, we infected activated cells with the wild-type strain or with those expressing either the toxin or the capsule. Both the fully virulent and the toxin-expressing strains were phagocytosed despite the large size of the *B. anthracis* bacilli (~5 microns) (Figure 3A and 3B). The capsule of the wild-type strain can clearly be distinguished in the inset of Figure 3A. Three hours post-infection, activated neutrophils killed 70% of the wild-type strain, more than 90% of the toxin-producing bacteria, and 80% of the capsule producers (Figure 3C). These data show that the capsulated strains are slightly more resistant to neutrophil killing at an MOI of 10:1. In contrast, at an MOI of 1:1 and a 30-min infection period, neither the toxin nor the capsule significantly affects the killing capacity of the neutrophils (Figure 3D).

To determine if extracellular mechanisms are responsible for *B. anthracis* killing, we again inhibited neutrophil phagocytosis. In the presence of cytochalasin D, all three *B. anthracis* strains were killed equally well (Figure 3D). This implies that extracellular mechanisms play an important role in the neutrophil-mediated killing. Interestingly, the proportion of intracellular killing was comparable in all three strains, indicating that neither the toxin nor the capsule has a pronounced antiphagocytic effect. These data are in agreement with O'Brien et al. [18] who showed that when the neutrophil is exposed to the toxin and the bacteria at the same time, the anthrax toxin does not inhibit phagocytosis. Although the capsule is an absolute prerequisite for virulence [19], the role of the capsule in the interaction of *B. anthracis* with host cells is not well understood [20,21]. It has been inferred, however, from studies with other capsulated microorganisms, that a capsule can affect phagocytosis. The data presented here show that, at least in the conditions tested, the *B. anthracis* capsule does not inhibit phagocytosis.

**Figure 4:**
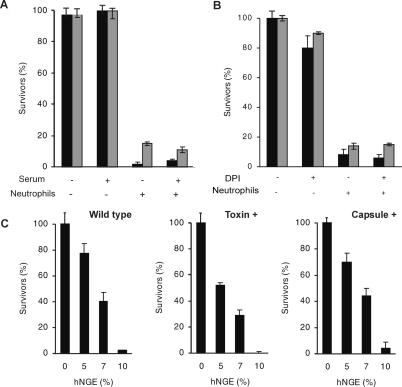
Human Neutrophils Efficiently Kill Vegetative *B. anthracis* Independently of Oxygen Radicals (A) Opsonization is not required for neutrophil anti-*B. anthracis* activity. PMA-activated neutrophils were infected with capsule (gray bar)- and toxin (black bar)-producing strains at an MOI of 1:1 in the presence or absence of serum for 30 min. (B) Neutrophils kill both capsule (gray bar)- and toxin (black bar)-producing *B. anthracis* independently of NADPH oxidase activity. Activated neutrophils were incubated with DPI (10 μM) before infection. Bacterial counts were determined 30 min post-infection (MOI 1:1). (C) hNGE kills *B. anthracis* at low concentrations. Wild-type, toxin-, and capsule-producing strains were incubated with 5%, 7%, and 10% hNGE. After 30 min, remaining CFUs were determined and referred to the number of bacteria in controls of bacteria incubated in buffer. Error bars show SD of three experiments.

Interestingly, opsonization did not significantly affect the rate of killing of either capsule- or toxin-expressing vegetative bacteria (Figure 4A) indicating that activated neutrophils might recognize *B. anthracis* directly. The independence of opsonization was seen in both phorbol 12-myristate 13-acetate (PMA) (Figure 4A) and IL-8-activated neutrophils (unpublished data). Interestingly, previous reports [20,22] showed that opsonization of vegetative bacilli had an effect on the generation of ROS, but bacterial killing was not directly tested.

Analogously to spore killing, vegetative *B. anthracis* are killed independent of ROS production (Figure 4B). These data imply that the described reduced ROS production in neutrophils of anthrax patients [22] might not play a significant role in vivo. Interestingly, these data are in contrast to the Gram-positive bacterium *Staphylococcus aureus,* which is killed in a ROS-dependent manner [23]. Therefore, we investigated whether *B. anthracis* is killed by granule antimicrobials. Indeed, all three *B. anthracis* strains were surprisingly susceptible to low concentrations of hNGE (Figure 4C). In comparison, 10% hNGE only killed 50% of a *S. aureus* culture (unpublished data). Corresponding to neutrophil killing, both capsulated strains were less susceptible to hNGE in comparison to the toxin-expressing strain.

### Neutrophils Kill *B*. *anthracis* through α-Defensins

**Figure 5:**
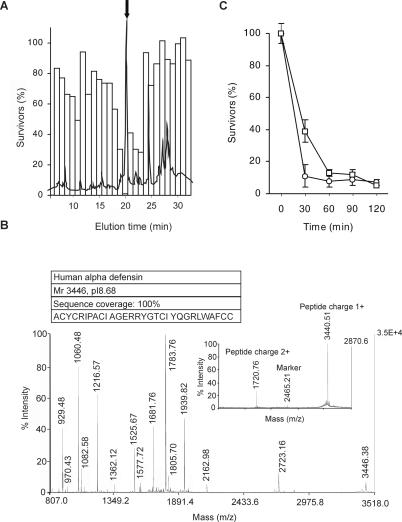
Neutrophils Kill *B. anthracis* through α-Defensins (A) Purification of the anti-*B.anthracis* activity in hNGE; hNGE was resolved in a C4 RP- HPLC column (A) and the fractions were tested for their killing activity of *B. anthracis* (bars). The main chromatographic peak coincides with the *B. anthracis* killing activity (arrow). **(**B) The active component was identified by MALDI MS PMF as human neutrophil α-defensins. Combined MASCOT search of PMF and MS/MS data (five MS/MS spectra) resulted in a Score value of 313. Peak heights of molecules not belonging to the identified protein were below 3%. Purity of sample was confirmed by the analysis of the uncleaved peptide (inset). As the protein was analyzed in non-reducing conditions it is present in its oxidized form reducing the Mr from 3,446 to 3,440. (C) The purified fraction and synthetic α-defensin have comparable specific activity. Vegetative toxin-producing bacteria were incubated for varying time points with 8 μg/ml synthetic (□) or purified (○) peptide and referred to the number of bacteria in buffer only. Error bars indicate SD of three experiments.

To identify the components of the hNGE responsible for *B. anthracis* killing, hNGE was fractionated by C4 reverse phase high-performance liquid chromatography (RP-HPLC). The peak fraction exhibiting antimicrobial activity in the first column (Figure 5A) was further purified in a C4 RP-HPLC column with a different mobile phase (see Materials and Methods) followed by a C18 RP-HPLC column. Tricine SDS-PAGE analysis of the antimicrobial fraction showed a peptide migrating at three to four kDa (unpublished data). The active component was identified by MALDI MS peptide mass fingerprinting (PMF) and MS/MS data of five peptides as human neutrophil α-defensin (Figure 5B). Only the 1-fold and the 2-fold charged molecule peaks were detected (Figure 5B, inset) in the MS spectrum of the non-trypsinized peptide, confirming purity of the sample. These results were verified by LC-ESI-MS analysis (unpublished data).

α-defensins are a group of small antimicrobial peptides found in human neutrophil granules [24]. The three major human α-defensins differ by one amino acid, and all kill bacteria [25] effectively, although some Gram-positive pathogens such as *S. aureus* are resistant [26]. To confirm that α-defensins are responsible for neutrophil killing of *B. anthracis,* we compared the antimicrobial activity of the purified fraction to synthetic human α-defensin (Figure 5C). These two samples had a comparable specific activity confirming human α-defensins as an hNGE component responsible for killing wild-type, toxin-producing, and capsule-producing (unpublished data) *B. anthracis* strains.

The data presented here show that human neutrophils engulf *B. anthracis* spores and induce them to germinate. Neutrophils then efficiently kill the bacteria independent of toxin or capsule expression. The timely recruitment of neutrophils to the site of a cutaneous anthrax infection could explain why these infections resolve spontaneously. In inhalation anthrax, where there is little neutrophil infiltration [11], the infection progresses unchecked and leads to sepsis and death. Here we also show that α-defensins play an important role in the potent anti-*B. anthracis* activity of neutrophils.

## Materials and Methods

### Human neutrophil isolation.

Heparinized venous blood was obtained after informed consent of healthy volunteers. Neutrophils ( > 95% pure) were isolated using a dextran-sedimentation protocol followed by centrifugation in a Ficoll-Paque gradient [27]. Erythrocytes were lysed with ultra-pure water for 30 s and cells washed in HBSS- and diluted in RPMI/10 mM HEPES with or without 10% FCS.

### Bacterial strains.

All experiments were performed using either the wild-type (Vollum), the capsule-producing (Pasteur), or toxin-producing (Sterne) *B. anthracis* strain and performed in a BSL-3 facility. The anthrax strains were a kind gift of W. Beyer, University of Hohenheim. For toxin expression, bacteria were grown on BHI agar plates with 0.8% sodium bicarbonate at 37 °C, 5% CO_2_. A single colony was subcultured overnight in the supplemented BHI. The wild-type strain was additionally incubated in 5% CO_2_ atmosphere. The capsule-producing strain was grown on BHI plates for 2 d at 37 °C and 20% CO_2_ and a single colony was subcultured overnight in RPMI supplemented with 10% BHI, 10 mM HEPES, and 0.8% sodium bicarbonate.

### Toxin and capsule production.

Lethal and edema factors, as well as protective antigen expressions, were monitored by Western Blot analysis of TCA-precipitated bacterial culture supernatant using antibodies from Abcam (Abcam, Cambridge, United Kingdom); γ-D-glutamic acid capsule expression was examined by India ink stain.

### Preparation of *B. anthracis* endospore suspensions.

Strains were grown in BHI overnight at 37 °C before plating on meat yeast agar and incubated for 10 d at 37 °C and 5% CO_2_. The spores were scraped off, washed five times with PBS, and kept in PBS at 4 °C until use. The wild-type strain spores were purified with Renografin as described by Ivins et al. [28]. They were washed four times with water and kept in water at 4 °C. Purity was confirmed by phase contrast microscopy and heat treatment (60 °C, 45 min).

### Antimicrobial activity of neutrophils.

2 × 10^6^/ml neutrophils were allowed to adhere to plastic plates for 15 min before activation with 25 nM PMA Sigma (Sigma, St. Louis, Missouri, United States) or 10 ng/ml Il-8 (BioCat, Heidelberg, Germany) for 30 min. Bacterial suspensions (vegetative cells or spores) were centrifuged onto the neutrophils (1,250 rpm, 10 min) and incubated for the indicated time points at 37 °C. For opsinization experiments vegetative bacteria were preincubated for 10 min with 5% non-inactivated human serum. Killing experiments were performed in the presence of a final concentration of 5% human serum. The cells were scraped off and serial dilutions plated on BHI agar plates. Colonies were counted after 12-h incubation at 37 °C, 5% CO_2_. Bacterial killing was measured as percentages of control values (bacteria incubated alone in media without neutrophils). To measure antimicrobial activity in the absence of ROS, neutrophils were preincubated with 10 μM of the NADPH oxidase inhibitor DPI [17] for 30 min. Inhibition of the respiratory burst was confirmed by enhanced chemiluminescence of PMA (25 nM)-activated neutrophils [29]. The experiment was read in a BD Pharmagen TM, Monolight 3096 microplate luminometer (BD Biosciences Pharmingen, San Diego, California, United States).

### Inhibition of phagocytosis.

Neutrophils were isolated as described above and allowed to adhere to plastic plates for 15 min. Cells were activated with PMA (25 ng/ml) for 30 min, spun down at 1,250 rpm for 5 min. The supernatant was discarded and media containing 10 μg/ml cytochalsin D (Sigma) was added for 15 min at 37 °C. Cells were infected with spores or vegetative bacteria as described above. To confirm that spores do not germinate in the presence of neutrophils, 25 μg/ml PenicillinG (Sigma-Aldrich) was added to the cytochalsin D- treated cells prior to infection.

### Chromatography.

hNGE was fractionated with a C4 RP-HPLC column (Vydac Protein C4 Column, 3.9 × 250 mm, 5 μm). Proteins were eluted with a gradient of increasing concentrations of acetonitril containing 0.1% (v/v) trifluoroacetic acid (flow rate 1 ml/min). Fractions were lyophilized, dissolved in 20 mM of sodium acetate buffer and tested for antimicrobial activity. The antimicrobial activity was further purified in a second C4 column, (mobile phase 100 mM of ammonium acetate [pH5]), followed by a C18 RP-HPLC column (X-terra RP18, 3.9 × 150 mm, 3.5 μm) with an acetonitril and 0.1% (v/v) trifluoroacetic acid gradient (flow rate 1 ml/min). Separation was performed in a Waters 626 LC System with a Water 996 Photodiode Array Detector (Waters, Milford, Massachusetts, United States).

### Bactericidal activity.

hNGE was prepared as described in [30]. The bactericidal activity of hNGE, HPLC fractions, or synthetic human α-defensin 2 (American Peptide Company, Sunnyvale, California, United States) was determined by incubation with bacteria (10^7^ bacteria/ml) in a casamino buffer (0.3% casamino acids, HBSS-, 10 mM HEPES [pH7.4]) at 37 °C, with the indicated doses, shaking for 30 min. Antimicrobial activity of each hNGE batch was tested with the toxin- expressing *B. anthracis* strain. Survivors were counted in serial dilutions. Bacterial killing was measured as percentages of control values (bacteria incubated in media alone).

### Electron microscopy.

For TEM, cells were fixed with 2.5% glutaraldehyde, post-fixed with 1% osmium tetroxide, contrasted with uranylacetate and tannic acid, and dehydrated and embedded in Spurr's (Ted Pella, Redding, California, United States). After polymerization, specimens were cut at 60 nm and contrasted with lead citrate. Specimens were analyzed in a Leo 906E TEM. For scanning electron microscopy: Cells were fixed with 2.5% glutaraldehyde, post-fixed using repeated incubations with 1% osmium tetroxide/1% tannic acid, dehydrated with a graded ethanol series, critical-point dried, and coated with 2 nm platinum. After dehydration and critical-point drying, the specimens were coated with 5 nm Platinum/Carbon and analyzed in a Leo 1550 scanning electron microscopy.

### Mass spectrometry.

The identity and purity of the antimicrobial component was analyzed by MALDI mass spectrometry (Proteomics 4700 workstation, Applied Biosystems, Foster City, California, United States), PMF, MS/MS analysis, and mass analysis of the uncleaved protein. The lyophilized sample was digested with 50 mM NH_4_HCO_3_, 5% acetonitrile, 2% (w/v) trypsin (Sequencing grade modified Trypsin, Promega, Madison, Wisconsin, United States), and 0.15M DTT for 4 h at 37 °C. The reaction was stopped with 0.2% TFA and mixed with matrix alpha-Cyano-4-hydroxycinnamic acid (CHCA) solubilized in 50% acetonitrile 0.3% TFA with a concentration of 5 mg/ml.

Analysis of PMFs was obtained with parameters: reflectron mode, 20 kV accelerating voltage, and a low mass gate of 800 Da. MS/MS spectra were obtained without collision gas. Parameters for database searches (MASCOT, http://www.matrixscience.com) were: 30 ppm peptide mass tolerance for PMF and 0.3 Da for MS/MS spectra. The uncleaved protein was analyzed in linear mode with CHCA as matrix with an internal marker (Mr 2465.21).

## Supporting Information

Figure S1 Inhibition of Respiratory Burst by DPI PMA-(25 nM) activated neutrophils were incubated in the presence (□) or absence (⋄) of DPI and chemiluminescence monitored in a BD Pharmagen TM, Monolight 3096 microplate luminometer in the presence of luminol.
(782 KB TIF)

## Abbreviations

CFU: colony forming unit
DPI: diphenyleneiodonium
hNGE: human neutrophil granule extract
MOI: multiplicity of infection
NADPH: nicotinamide adenosine dinucleotide phosphate
PMA: phorbol 12-myristate 13-acetate
PMF: peptide mass fingerprinting
ROS: reactive oxygen species production
RP-HPLC: reverse phase high-performance liquid chromatography
TEM: transmission electron microscopy
